# Individual development of preschool children-prevalences and determinants of delays in Germany: a cross-sectional study in Southern Bavaria

**DOI:** 10.1186/1471-2431-12-188

**Published:** 2012-12-05

**Authors:** Heribert L Stich, Bernhard Th Baune, Riccardo N Caniato, Rafael T Mikolajczyk, Alexander Krämer

**Affiliations:** 1 Department of Public Health, District of Erding, Erding, Germany; 2 Department of Public Health Medicine, School of Public Health, University of Bielefeld, Bielefeld, Germany; 3 School of Medicine, Discipline of Psychiatry, University of Adelaide, Adelaide, Australia; 4 Belgian Gardens Specialist Centre, Townsville, Queensland, Australia; 5 Bremen Institute for Prevention Research and Social Medicine, University of Bremen, Achterstr. 30, 28359 Bremen, Germany; 6 Helmholtz Centre for Infection Research, Braunschweig, Germany; 7 Hannover Medical School, Hannover, Germany

## Abstract

**Background:**

Even minor abnormalities of early child development may have dramatic long term consequences. Accurate prevalence rates for a range of developmental impairments have been difficult to establish. Since related studies have used different methodological approaches, direct comparisons of the prevalence of developmental delays are difficult. The understanding of the key factors affecting child development, especially in preschool aged children remains limited. We used data from school entry examinations in Bavaria to measure the prevalence of developmental impairments in pre-school children beginning primary school in 1997–2009.

**Methods:**

The developmental impairments of all school beginners in the district of Dingolfing- Landau, Bavaria were assessed using modified “Bavarian School Entry Model” examination from 1997 to 2009 (N=13,182). The children were assessed for motor, cognitive, language and psychosocial impairments using a standardised medical protocol. Prevalence rates of impairments in twelve domains of development were estimated. Using uni- and multivariable logistic regression models, association between selected factors and development delays were assessed.

**Results:**

The highest prevalence existed for impairments of pronunciation (13.8%) followed by fine motor impairments (12.2%), and impairments of memory and concentration (11.3%) and the lowest for impairments of rhythm of speech (3.1%). Younger children displayed more developmental delays. Male gender was strongly associated with all developmental impairments (highest risk for fine motor impairments = OR 3.22, 95% confidence interval 2.86-3.63). Preschool children with siblings (vs. children without any siblings) were at higher risk of having impairments in pronunciation (OR 1.31, 1.14-1.50). The influence of the non-German nationality was strong, with a maximum risk increase for the subareas of grammar and psychosocial development. Although children with non-German nationality had a reduced risk of disorders for the rhythm of speech and pronunciation, in all other 10 subareas their risk was increased.

**Conclusions:**

In preschool children, most common were delays of pronunciation, memory and concentration. Age effects suggest that delays can spontaneously resolve, but providing support at school entry might be helpful. Boys and migrant children appear at high risk of developmental problems, which may warrant tailored intervention strategies.

## Background

Despite exposure to a variety of risk factors, most preschool children do not show delays in physical, mental and emotional development [1,2]. Accurate prevalence rates for a range of developmental impairments have been difficult to establish and comparisons across studies are hampered by the use of different methodologies. However, there is some evidence that the prevalence of child developmental delays has increased over recent years [3]. Developmental deficits at the time of school enrolment were also linked to a later success in school. For example Duncan et al. [4] demonstrated in a meta-analysis of six studies that the levels of development at the school entry in math, reading and attention skills were the strongest predictors of later success in school. Identifying developmental delays prior to school entry and providing appropriate interventions can likely help improve school outcomes.

In German speaking countries, the term “developmental delay” has a broad meaning [5,6]. In contrast, experts in English speaking countries tended to focus mainly on motor and cognitive deficits when assessing developmental delays, while disorders of speech, emotion and psychological development were often not included or considered [7-9]. Furthermore, even within Germany developmental disorders were generally not classified consistently, and there are large variations among the studies that have been published to date [10-20].

In 2008, our group published data on rates of childhood impairments in consecutive cohorts of Bavarian children entering school from 1997 to 2005 [21]. The primary aim of the previous analysis was to describe and compare prevalences of disorders of development over time. In the current analysis, we use data from thirteen years (1997–2009) and extend our previous findings by analysing additional factors influencing development.

## Methods

Before entering primary school, all children in the federal state of Bavaria in Germany are examined by the local Child and Youth Health Services in the so-called school-enrolment-examination [22,23]. The data for all children entering school in the District of Dingolfing-Landau in Bavaria has been collected for the years 1997–2009. The assessment consisted of a comprehensive physical, psychological and behavioural assessment that has been developed by the Public Health Service of Bavaria. The examination was designed to provide information to the department of education for the purpose of facilitating changes in education policy and to allow early intervention for children at risk [24]. The assessment was based on a modified manual established in 1997 by the Working Group “School and Youth Health Care in Public Health Service” [25]. Four areas with twelve associated subareas of individual delays were defined (Table 1). The inability to pass a specific test was classified as a developmental delay.

**Table 1:** **Assessment of development of preschool children based on** “**Bavarian School Entry Model**” *

	**Main areas of skills**	**Subareas of skills**	**Tests**
Biomedical assessment	motor	gross motor	standing on one leg, jumping on one leg, going like a rope dancer, going with clapping hands
		fine body coordination	finger- opposition-test, drawing different figures and of persons
		grapho-motor coordination	
	speech	pronunciation	repeating words
		grammar	retelling a short story, explaining rules of a known game
		rhythm of speech	repeating longer sentences
	cognition	memory and concentration	repeating sentences with 7–10 words including three adjectives; repeating four single numbers in correct sequences
		perseverance	discontinuity of capacity to attend during the examination
		abstraction	building pairs, finding a subject of various objects belonging together
		visual perception	reception and knowing of simple geometric figures or silhouettes of figures and animals
		arithmetic	counting form 1 to 10 in correct sequences
Psychological assessment	psychosocial	behaviour	erratic, overly bonded mother (no separation possible during examination?), hostility towards examiner
		emotionality	major mood
		psycho- motor	agitation, inability of sitting calmly during examination

* tests based on so called “milestones of development” [36-38].

Diagnosis and documentation of delays forming base for the current study was conducted by the investigation team of School Health Services in the District of Dingolfing-Landau. During the whole study period this team remained unchanged. The use of routinely collected and anonymised data for the current analysis was reviewed by the ethic committee of the University of Bremen and exemption was granted.

### Statistical analysis

We used the software package SPSS 17.0 to calculate the prevalence of developmental delays. Using uni- and multivariable logistic regression models, crude and adjusted associations between developmental delays and selected factors were determined. To assess the influence of age, three age groups (up to 5.49 years vs. between 5.50 to 6.5 years vs. 6.51 years and older) were defined. Sex, nationality and having siblings (any versus no) were entered as binary variables in the models.

## Results

### Study population

During the 13 years of observation period, 13,279 preschool children were examined. For 97 of them, the data was incomplete, so the final study sample contained 13,182 children. The median age was 5.93 years (standard deviation (SD) +/−0.39) and there were 51.7% boys and 48.3% girls. The majority of children had German nationality (88.7%) and most had at least one sibling (79.8%).

### Prevalence of developmental impairments

We found the highest prevalence of impairment in the area of pronunciation (13.8%), followed by fine motor impairments (12.2%), and impairments of memory and concentration (11.3%) (Table 2). In contrast, very low rates of impairments existed for rhythm of speech (3.1%) and abstraction (3.2%). The percentages of children with developmental delays for the remaining seven subareas of delays were between 4.0% (for calculation) and 9.7% (for grapho-motor coordination).

**Table 2:** General and stratified prevalences of delays

**Delays of development**	**Prevalences**	**Age**			**Sex**		**Nationality**		**Having siblings**	
		**Below 5.49 years**	**Between 5.50 and 6.50 years**	**6.51 years or older**	**Girl**	**Boy**	**German**	**Non- German**	**No**	**Yes**
Gross motor skills	6.1% (799/13088) m=191	6.4% (108/1693)	6.0% (635/10532)	6.5% (56/863)	3.4% (217/6325)	8.6% (582/6763)	5.9% (683/11559)	7.8% (115/1474)	5.6% (139/2501)	6.2% (633/10190)
Fine motor coordination	12.2% (1592/13087) m=192	21.0% (355/1693)	11.1% (1170/10532)	7.8% (67/862)	6.3% (396/6325)	17.7% (1196/6762)	11.6% (1346/11558)	16.4% (242/1474)	11.4% (285/2500)	12.2% (1247/10190)
Grapho-motor coordination	9.7% (1269/13087) m=192	17.1% (290/1693)	8.8% (925/10532)	6.3% (54/862)	5.0% (317/6325)	14.1% (952/6762)	9.1% (1053/11558)	14.5% (213/1474)	9.3% (232/2500)	9.7% (993/10190)
Pronunciation	13.8% (1810/13087) m=192	15.1% (256/1693)	13.7% (1442/10532)	13.0% (112/862)	9.5% (604/6325)	17.8% (1206/6762)	14.3% (1653/11558)	10.2% (151/1474)	11.3% (282/2500)	14.5% (1474/10190)
Grammar	4.0% (519/13087) m=192	4.9% (83/1693)	3.7% (390/10532)	5.3% (46/862)	2.8% (176/6325)	5.1% (343/6762)	3.4% (389/11558)	8.8% (130/1474)	3.9% (98/2500)	3.9% (402/10190)
Rhythm of speech	3.1% (403/13087) m=192	3.1% (52/1693)	3.0% (317/10532)	3.9% (34/862)	2.0% (127/6325)	4.1% (276/6762)	3.2% (370/11558)	2.1% (31/1474)	3.0% (76/2500)	3.1% (317/10190)
Memory and concentration	11.3% (1483/13086) m=193	18.7% (317/1693)	10.3% (1088/10531)	9.0% (78/862)	9.5% (598/6324)	13.1% (885/6762)	10.3% (1189/11557)	19.5% (288/1474)	11.3% (283/2500)	11.1% (1132/10189)
perseverance	6.8% (892/13086) m=193	12.7% (215/1693)	5.9% (624/10531)	6.1% (53/862)	5.6% (352/6324)	8.0% (540/6762)	6.2% (716/11557)	11.6% (171/1474)	7.1% (178/2500)	6.6% (669/10189)
abstraction	3.2% (425/13086) m=193	3.7% (63/1693)	3.1% (329/10531)	3.8% (33/862)	2.4% (151/6324)	4.1% (2274/6762)	2.8% (319/11557)	7.1% (105/1474)	3.4% (84/2500)	3.2% (327/10189)
visual perception	4.4% (571/13086) m=193	4.4% (74/1693)	4.3% (448/10531)	5.7% (49/862)	2.8% (179/6324)	5.8% (392/6762)	3.7% (432/11557)	9.3% (137/1474)	4.5% (112/2500)	4.2% (433/10189)
arithmetic	4.0% (527/13087) m=192	5.0% (84/1693)	3.9% (411/10532)	3.7% (32/862)	3.4% (215/6325)	4.6% (312/6762)	3.4% (397/11558)	8.5% (125/1474)	3.8% (96/2500)	3.9% (402/10190)
psychosocial development	6.7% (874/13087) m=192	11.6% (196/1693)	5.9% (621/10532)	6.6% (57/862)	5.6% (352/6325)	7.7% (522/6762)	6.2% (712/11558)	10.3% (152/1474)	7.5% (188/2500)	6.5% (659/10190)

m= missing.

Younger children (<5.5 years) demonstrated the highest rates of fine motor impairments (21.0%), compared to 11.1% and 7.8% for the intermediate (5.5-6.5 years) and older age groups (>6.5 years) respectively. For most impairments, increasing age reduced rates of delays, with the exception of visual perception, abstraction, rhythm of speech and grammar for which older children had higher rates of development delays.

Boys had significantly higher rates of impairments in all subareas of development. Some differences were found between children of German nationality and those with a non-German nationality: children with migration background had higher rates of impairment in nearly all areas of development, except for pronunciation and rhythm of speech. We also found differences between children with and without siblings. Children with no siblings did better in the areas of motor functioning and pronunciation, but worse in psychosocial development (Table 2).

### Factors associated with the presence of developmental delays (uni- and multivariable analysis)

In comparison to the reference group of the youngest children, the risk was reduced by 22% and 68% for age groups 5.50-6.50 years and 6.51 years or older in subareas of fine motor coordination, grapho-motor coordination, grammar, memory and concentration, arithmetic and psychosocial development (Table 3). This age-specific effect increased to a maximum of 73% for the subarea of fine motor coordination in adjusted model (Table 4).

**Table 3:** **Association of selected risk factors with delays of development** (**univariable logistic regression**, **Odds Ratios and 95**%-**confidence intervals**, **significant associations in bold**)

	**Age**					**Sex**			**Nationality**			**Having siblings**		
**Delays of development**	**Below 5.49 years**	**Between 5.50 and 6.50 years**	**p-value**	**6.51 years or older**	**p-value**	**Girl**	**Boy**	**p-value**	**German**	**Non- German**	**p-value**	**No**	**Yes**	**p-value**
Gross motor skills	1	0.94 (0.76-1.16)	0.57	1.02 (0.73-1.42)	0.92	1	**2**.**65** (**2**.**26**-**3**.**11**)	<**0**.**0001**	1	**1**.**35** (**1**.**10**-**1**.**66**)	**0**.**004**	1	1.13 (0.93-1.36)	0.22
Fine motor coordination	1	**0**.**47** (**0**.**41**-**0**.**54**)	<**0**.**0001**	**0**.**32** (**0**.**24**-**0**.**42**)	<**0**,**0001**	1	**3**.**22** (**2**.**86**-**3**.**63**)	<**0**.**0001**	1	**1**.**49** (**1**.**28**-**1**.**73**)	<**0**.**0001**	1	1.08 (0.95-1.24)	0.25
Grapho- motor coordination	1	**0**.**47** (**0**.**40**-**0**.**54**)	<**0**.**0001**	**0**.**32** (**0**.**24**-**0**.**44**)	<**0**.**0001**	1	**3**.**11** (**2**.**72**-**3**.**54**)	<**0**.**0001**	1	**1**.**69** (**1**.**44**-**1**.**97**)	<**0**.**0001**	1	1.06 (0.91-1.23)	0.48
Pronunciation	1	0.89 (0.77-1.03)	0.12	0.84 (0.66-1.07)	0.15	1	**2**.**06** (**1**.**85**-**2**.**28**)	<**0**.**0001**	1	**0**.**68** (**0**.**57**-**0**.**82**)	<**0**.**0001**	1	**1**.**33** (**1**.**16**-**1**.**52**)	<**0**.**0001**
Grammar	1	**0**.**75** (**0**.**59**-**0**.**95**)	**0**.**02**	1.09 (0.76-1.58)	0.64	1	**1**.**87** (**1**.**55**-**2**.**25**)	<**0**.**0001**	1	**2**.**78** (**2**.**26**-**3**.**41**)	<**0**.**0001**	1	1.01 (0.80-1.26)	0.95
Rhythm of speech	1	0.98 (0.73-1.32)	0.89	1.30 (0.83-2.01)	0.25	1	**2**.**01** (**1**.**68**-**2**.**57**)	<**0**.**0001**	1	**0**.**65** (**0**.**45**-**0**.**94**)	**0**.**02**	1	1.02 (0.79-1.32)	0.86
Memory and concentration	1	**0**.**50** (**0**.**44**-**0**.**57**)	<**0**.**0001**	**0**.**43** (**0**.**33**-**0**.**56**)	<**0**.**0001**	1	**1**.**44** (**1**.**29**-**1**.**61**)	<**0**.**0001**	1	**2**.**18** (**1**.**84**-**2**.**44**)	<**0**.**0001**	1	0.98 (0.85-1.12)	0.77
Perseverance	1	**0**.**40** (**0**.**37**-**0**.**51**)	<**0**.**0001**	**0**.**45** (**0**.**33**-**0**.**62**)	<**0**.**0001**	1	**1**.**47** (**1**.**28**-**1**.**69**)	<**0**.**0001**	1	**1**.**99** (**1**.**67**-**2**.**37**)	<**0**.**0001**	1	0.92 (0.77-1.09)	0.32
Abstraction	1	0.83 (0.63-1.10)	0.20	1.03 (0.67-1.58)	0.89	1	**1**.**73** (**1**.**41**-**2**.**11**)	<**0**,**0001**	1	**2**.**70** (**2**.**15**-**3**.**39**)	<**0**.**0001**	1	0,95 (0.75-1.22)	0,70
Visual perception	1	0.97 (0.76-1.25)	0.83	1.32 (0.91-1.91)	0.14	1	**2**.**11** (**1**.**76**-**2**.**53**)	<**0**.**0001**	1	**2**.**64** (**2**.**16**-**3**.**22**)	<**0**.**0001**	1	0.95 (0.77-1.17)	0.61
Arithmetic	1	**0**.**78** (**0**.**61**-**0**.**99**)	**0**.**04**	0.74 (0.49-1.12)	0.15	1	**1**.**38** (**1**.**15**-**1**.**64**)	<**0**.**0001**	1	**2**.**61** (**2**.**11**-**3**.**21**)	<**0**.**0001**	1	1.03 (0.82-1.29)	0.81
Psychosocial development	1	**0**.**48** (**0**.**40**-**0**.**57**)	<**0**.**0001**	**0**.**54** (**0**.**40**-**0**.**74**)	<**0**.**0001**	1	**1**.**42** (**1**.**23**-**1**.**63**)	<**0**.**0001**	1	**1**.**73** (**1**.**44**-**2**.**08**)	<**0**.**0001**	1	0.85 (0.72-1.01)	0.06

1=reference.

**Table 4:** **Association of selected risk factors with delays of development** (**Multivariable logistic regression** - **adjusted Odds Ratios and 95**%- **confidence intervals**, **significant associations in bold**)

	**Age**					**Sex**			**Nationality**			**Having siblings**		
**Delays of development**	**Below 5.49 years**	**Between 5.50 and 6.50 years**	**p-value**	**6.51 years and older**	**p-value**	**Girl**	**Boy**	**p-value**	**German**	**Non- German**	**p-value**	**No**	**Yes**	**p-value**
Gross motor skills	1	0.89 (0.72-1.10)	0.28	0.92 (0.65-1.30)	0.63	1	**2**.**73** (**2**.**32**-**3**.**22**)	<**0**.**0001**	1	**1**.**34** (**1**.**08**-**1**.**66**)	**0**.**01**	1	1.13 (0.93-1.37)	0.21
Fine motor coordination	1	**0**.**43** (**0**.**37**-**0**.**49**)	<**0**.**0001**	**0**.**28** (**0**.**21**-**0**.**38**)	<**0**.**0001**	1	**3**.**37** (**2**.**98**-**3**.**81**)	<**0**.**0001**	1	**1**.**41** (**1**.**20**-**1**.**65**)	<**0**.**0001**	1	1.09 (0.95-1.26)	0.22
Grapho- motor coordination	1	**0**.**42** (**0**.**36**-**0**.**49**)	<**0**.**0001**	**0**.**30** (**0**.**22**-**0**.**40**)	<**0**.**0001**	1	**3**.**27** (**2**.**85**-**3**.**74**)	<**0**.**0001**	1	**1**.**60** (**1**.**35**-**1**.**89**)	<**0**.**0001**	1	1.07 (0.92-1.25)	0.40
Pronunciation	1	**0**.**83** (**0**.**72**-**0**.**97**)	**0**.**02**	0.78 (0.61-1.00)	0.05	1	**2**.**09** (**1**.**88**-**2**.**32**)	<**0**.**0001**	1	**0**.**66** (**0**.**55**-**0**.**79**)	<**0**.**0001**	1	**1**.**31** (**1**.**14**-**1**.**50**)	<**0**.**0001**
Grammar	1	0.77 (0.60-0.98)	0.58	1.39 (0.89-2.15)	0.15	1	**1**.**79** (**1**.**45**-**2**.**21**)	<**0**.**0001**	1	**2**.**52** (**1**.**95**-**3**.**26**)	<**0**.**0001**	1	1.04 (0.81-1.34)	0.76
Rhythm of speech	1	0.90 (0.66-1.21)	0.47	1.16 (0.74-1.81)	0.52	1	**2**.**09** (**1**.**69**-**2**.**60**)	<**0**.**0001**	1	**0**.**61** (**0**.**41**-**0**.**90**)	**0**.**01**	1	1.01 (0.78-1.30)	0.97
Memory and concentration	1	**0**.**49** (**0**.**43**-**0**.**57**)	<**0**.**0001**	**0**.**42** (**0**.**31**-**0**.**56**)	<**0**.**0001**	1	**1**.**51** (**1**.**33**-**1**.**71**)	<**0**.**0001**	1	**2**.**01** (**1**.**73**-**2**.**33**)	<**0**.**0001**	1	0.99 (0.85-1.16)	0.90
Perseverance	1	**0**.**42** (**0**.**36**-**0**.**50**)	<**0**.**0001**	**0**.**44** (**0**.**32**-**0**.**61**)	<**0**.**0001**	1	**1**.**53** (**1**.**32**-**1**.**76**)	<**0**.**0001**	1	**1**.**82** (**1**.**51**-**2**.**19**)	<**0**.**0001**	1	0.93 (0.79-1.11)	0.44
Abstraction	1	0.86 (0.65-1.14)	0.29	1.07 (0.67-1.66)	0.77	1	**1**.**77** (**1**.**44**-**2**.**17**)	<**0**.**0001**	1	**2**.**76** (**2**.**18**-**3**.**48**)	<**0**.**0001**	1	0.99 (0.78-1.27)	0.95
Visual perception	1	1.01 (0.78-1.31)	0.96	1.33 (0.90-1.96)	0.15	1	**2**.**13** (**1**.**77**-**2**.**56**)	<**0**.**0001**	1	**2**.**65** (**2**.**15**-**3**.**27**)	<**0**.**0001**	1	0.98 (0.79-1.22)	0.87
Arithmetic	1	0.79 (0.62-1.01)	0.06	0.73 (0.47-1.12)	0.15	1	**1**.**40** (**1**.**16**-**1**.**68**)	<**0**.**0001**	1	**2**.**56** (**2**.**06**-**3**.**18**)	<**0**.**0001**	1	1.07 (0.85-1.34)	0.57
Psychosocial development	1	**0**.**48** (**0**.**40**-**0**.**57**)	<**0**.**0001**	**0**.**52** (**0**.**38**-**0**.**71**)	**0**.**001**	1	**1**.**47** (**1**.**28**-**1**.**70**)	<**0**.**0001**	1	**1**.**66** (**1**.**38**-**2**.**01**)	<**0**.**0001**	1	0.86 (0.73-1.02)	0.08

1=reference.

With the lowest risk increase of 42% for psychosocial development and the highest risk increase of 222% for fine motor coordination, male gender was a strongest risk factor among those analysed for all subareas of delays in individual development (Table 3). With a maximum increase of risk in the subarea of fine motor coordination (45%) and grapho-motor coordination (16%) the risk increased even further in multivariable regression analysis. Only for grammar, there was a decreased risk of impairment found in boys (Table 4).

The influence of the non-German nationality was strong, with a maximum risk increase of 178% for the subarea of grammar and 173% for psychosocial development. Although children with non-German nationality had a reduced risk of disorders for the rhythm of speech (a 35% protection effect) and pronunciation (32%), in all other 10 subareas their risk was increased (Table 3). Only minimal differences were seen between crude and adjusted associations in all subareas of development delays for the influence of nationality (Table 4).

The presence or absence of siblings had no important influence on the studied developmental impairments. Some weak positive and negative effects were identified, but these did not reach statistical significance. The one exception was a the increased risk for disorders of pronunciation for children with siblings (OR 1.31, 1.14-1.50).

## Discussion

In international comparison, school entrance examinations differ in their form and implementation from country to country. Because of methodical differences, a direct comparison of impairments reported in different studies is difficult. Previous research has tended to focus on only one or two areas of delays rather than conducting a complete assessment of multiple dimensions [7-20,26-31]. In this respect, our study provides a more comprehensive perspective.

### Strengths and limitations of the study

The major strength of this study is the large, locally representative sample from a clearly defined geographical area. Since examinations used for assessment of developmental delays were mandatory before school enrolment, the selection bias was minimised. Furthermore, the assessment of developmental delays was conducted by a single team using standardised methods, preventing inter-observer variation. However, there are also several limitations. Data from the studied population might not be representative for the whole Germany – in fact one can expect differences based on variation in social status and local ethnicity mix. Furthermore, we were not able to assess the parental socio-economic status, which is potentially an important confounder of childhood development. Also, we were not able to study the consequences of the observed delays for the long term development. This should be subject of additional studies in the future.

### Prevalence of development delays

In 2003, a taxonomy and assessment protocol for developmental delays was published by American medical specialists [8]. Based on this classification of “global development delays”, there were the domains of “gross/fine motor”, “speech/language”, “cognition”, “social/personal” and “activities of daily living” [7,30,31]. The nomenclature in our study was based on the first four “domains”, but used twelve “subdomains” of development following the local German conventions.

In our study population, approximately every 16^th^ child showed impairments in “gross motor skills”, every 8^th^ child in “fine motor coordination” and slightly less than every 10^th^ child in “grapho-motor coordination”.The rates of delays observed in our study for gross motor coordination were lower and for “fine and grapho-motor coordination” were higher than the rates for the whole of Bavaria. For comparison, 84.7% of 129,597 preschool children in Bavaria in 2004–2005 were able to stand on one leg (a test for gross motor coordination) and 90.8% were able to make a regular hand-coordination-test (a test for fine- and grapho-motor development) [32]. Similar prevalences were found in health reports from Baden-Württemberg (in 1998: 6.5% for gross motor coordination, 4.5% for fine body coordination and 7.5% for grapho-motor coordination) [16] and from Hesse (disorders of “coordination” for the years 1998–2005 on average around 8.5%) [13]. It is interesting that according to the results of the Early Childhood Longitudinal Study Birth Cohort (ECLS-B), in which children born in 2001 were assessed with respect to their development by three consecutive surveys over six years (preschool wave 2005/06; kindergarten wave 2006 and 2007), partly a significantly higher prevalence of impaired gross motor skills was found [33]. This difference in the prevalence between the Bavarian and U.S. children may have been caused mainly by different study designs: American children were examined methodically in a highly differentiated way, while the school entrance examinations in Germany were just screening examinations.

Problems of pronunciation were the most common developmental delay in our study. In the domain of “speech”, delays in “grammar” and “rhythm of speech” were less common, with all other types of impairment being far less frequent. Comparatively lower rates for disorders of language (1999: 0.4% in “rhythm of speech” and 2.2% for “dysgrammatism”) were found for the State of Baden-Württemberg in previous studies [16]. In contrast, in Hesse, significantly higher prevalences were observed, with approximately 11%-15% of the children having delays in speech in the cohorts of 1998–2005 [13]. The main reason for differences may be the different tests used in school entry examinations in the various states of Germany.

The high prevalence of over 10% for delays of memory and concentration was unexpected, whereas the frequencies of 3.2% to 6.8% for the remaining four subareas of cognitive development were not so surprising given previous findings [1]. Our findings are in clear contrast to reports from the City of Bonn for 2001–2005 (2001: 23.6% vs. 2002: 27.5% vs. 2003:18.9% vs. 2004: 23.2% vs. 2005: 16.3%) [10], and for the District of Mettmann for both years 2000 and 2003 (20.1% vs. 22.2%) for the subarea of “visual perception” [15]. Generally, the investigation of cognitive delays in preschool children did not receive sufficient attention over a long time period [1]. Only recently the situation is changing [29].

We identified problems of psychosocial development in every 15^th^ child in our survey. Until recent years, there appears to have been little interest in the assessment of psychosocial impairments. To some degree, psychosocial adjustment has been seen as a vague criterion, difficult to operationalize and not suitable for objective quantification. Nonetheless, it has become clear that it forms an important part of child development, and attempts to assess it are important [34]. Substantial differences were found in the rates of psychosocial problems in other regions in Germany in data published by Public Health Services. In the State of Hesse, 4.5%-5.0% of children had disorders of psychosocial functioning [13], compared to 8.3% for the State of Schleswig-Holstein and 13.6% in the District of North Friesland [14]. Thus, our prevalence figures are on the lower end in comparison with other regions in Germany. However, different methodologies and diagnostic approaches make direct comparison of these figures difficult. Developing more uniform and standardised assessment tools and diagnostic categories would be important to collate data from different regions and countries, and effectively utilise it for research and public health interventions.

### Factors associated with development delays

Many factors negatively affecting child development have been identified in the literature, however the precise magnitude of effects remains difficult to establish. Some attempts to measure these influences have been made over recent years. The effect of visiting a kindergarten [24], the influence of migration status [35] and the effects of selected behavioural and environment-related factors [1] on the development of preschool children were estimated. However, since these studies were all from the district of Dingolfing-Landau, it is not clear to what degree the same factors operate in other regions of Germany or in other countries.

Interesting aspect are the effects of age. The basis for assessing the level of development were the so-called milestones of child development [36]. These developmental milestones describe the acquisition of skills up to a defined age of the child [37,38], and 95% of children in the targeted age group are expected to have these skills. Given the reference to age, the younger children at the school entry examination might not developed yet the required skills [5]. Children with many or substantial delays will not be recommended for school entrance, but rather asked to delay the start of school for one year. Still, some children with developmental delays enter school and this is the group which would potentially benefit from interventions following school entry examination. In contrast, higher risks for the oldest group were rather surprising. One possible explanation is that there was some selection bias and those who were older at examination were not included in an earlier examination due to a perceived immaturity. In such case, the developmental delays identified in this group might indicate a more serious problem than among the younger children who might just be too young at the time point of examination and obtained the required skills shortly thereafter.

The strongest and consistent association we detected was related to the effect of sex on development. More boys than girls were affected by disorders of development in all areas we tested. This higher prevalence for delays in boys has been a consistent finding in the literature on childhood development [2]. A sex ratio of 1 to 2–4 for girls to boys has been already described for most developmental disorders in the previous analysis using a subset of the current data [1]. Our current analysis provided additional insights into how sex affects specific subareas of functioning. Male gender increased the risk of developmental impairments by 38%-222% for all subareas of development. In addition, only small changes of this effect were found in the adjusted regression models. The exact reasons for this sex differences in development have not yet been established, though many theories have been proposed [39].

In previous research, we studied the effect of having migrant status (non-German nationality) on developmental disorders [35]. We showed that more children with a non-German nationality demonstrated delays in motor, cognition and psychosocial development than other children. The elevated risk for preschool children with a non-German nationality in the current study in ten of twelve subareas of development was not unexpected. However, this indicated that among migrant children there can be specific stressors and developmental impediments that warrant further exploration and possibly tailored interventions. No doubt, one important reason for this observation can be a language barrier, which may be an important risk factor for some developmental delays, but may also make the assessment process biased.

In the past, primarily psychologists analysed the subject of the influence of siblings on child development [40,41]. Until a few years ago, no significant interest was paid from the public health point of view to the influence that the presence or absence of siblings has on child development in this age group. Thus, only a small number of reports in public health area can be identified which explored this issue [14,32], but the specific effect on the prevalence of developmental delays in the target group of school beginners has not been examined in a quantitative way as done in this study. Having siblings was not associated with most areas of development apart from pronunciation difficulties. This finding persisted despite a simultaneous adjustment for migration background. This contrasts with a study by Minnett et al. who found positive influence of siblings on development of children [42]. Our findings were possibly affected by the fact that we could not adjust for socio-economic status.

School entrance examinations were carried out during the period from November to April of calendar year. New school year in Bavaria begins always in the middle of following September. Thus, the preschoolers were assessed on average six months before entering primary school. During this period there is enough time for support measures if developmental delays were diagnosed in school entry examination. In this context, it should be noted that school entry examination is the only mandatory testing in the Federal Republic of Germany for children, but no standardised intervention measures are implemented for those who do not pass the examination or display deficits. For children with developmental deficits a specialized treatment can be recommended on individual basis, for example speech therapy, occupational therapy or supportive courses in sports.

## Conclusions

Data from our study suggests that developmental delays of speech and cognition are particularly common in school entry examinations. In particular, boys and migrant children appear at high risk of developmental problems, which may warrant tailored intervention strategies.

## Competing interests

The authors declare that they have no competing interests.

## Authors’ contributions

HLS has made substantial contributions to conception and design, has examined the children, analysed and interpreted data and drafted the manuscript. BTB has made substantial contributions to conception and design, analysed and interpreted data, has been involved in drafting the manuscript and revising it critically for important intellectual content. RNC has made substantial contributions to the writing of the manuscript. RTM supervised the statistical analysis and has been involved in revising the manuscript critically for important intellectual content. AK has been involved in drafting the manuscript or revising it critically for important intellectual content. All authors have given final approval of the final version of the manuscript.

## Pre-publication history

The pre-publication history for this paper can be accessed here:

http://www.biomedcentral.com/1471-2431/12/188/prepub

## References

[B1] Stich HL Disorders in preschool children. A differentiated analysis of prevalences of development delays over a ten-year period Kinder- und Jugendmedizin 2009 9 42 48

[B2] Wohlfeil A Developmental delay of school beginners with resulting partial performance limitations Offentl Gesundheitswes 1991 53 175 180 1714068

[B3] Flender J Early detection of children with defined disturbances Kinder- und Jugendarzt 2005 36 154 159

[B4] Duncan GJ Dowsett CJ Claessens A Magnuson K Huston AC Klebanov P Pagani LS Feinstein L Engel M Brooks-Gunn J School readiness and later achievement Dev Psychol 2007 43 1428 1446 18020822 10.1037/0012-1649.43.6.1428

[B5] Karch D Treatment of developmental disorders--principles, methods and indication Offentl Gesundheitswes 1990 52 491 495 1700352

[B6] Jäger-Roman E Impairment of development Supplement abstracts Weimar “Makes school ill” 2000 18

[B7] Fenichel GM Psychomotor retardation and regression 2001 4 Philadelphia: WB Saunders

[B8] Shevell M Ashwal S Donley D Flint J Gingold M Hirtz D Majnemer A Noetzel M Sheth RD Practice parameter: evaluation of the child with global developmental delay: report of the Quality Standards Subcommittee of the American Academy of Neurology and The Practice Committee of the Child Neurology Society Neurology 2003 60 367 380 10.1212/01.WNL.0000031431.81555.16 12578916

[B9] Sonnander K Early identification of children with developmental disabilities Acta Paediatr Suppl 2000 89 17 23 11055313 10.1111/j.1651-2227.2000.tb03091.x

[B10] City of Bonn Health state of preschool children 2005. Selected results of the Bonn School Entry Examination 2006 16 19

[B11] District of Ennepe-Ruhr Child and Youth Health Report 2006

[B12] Free Hanseatic City of Bremen Vulnerable childhood. Effects of social inequality on the opportunities for development of children in Bremen 2007 25 26

[B13] Social Ministry of Hesse Hessian Child and Youth Health Report 2006

[B14] District of Nordfriesland Child and youth health in the District of Nordfriesland 2006 15 17

[B15] District of Mettmann Report of child welfare 2004. Results of School Entry- and dental Examinations 2004 13 20

[B16] Landeshauptstadt Stuttgart unit Social Affairs YaHG Stuttgart 2000 health report 2000 109 116

[B17] District of Rhein-Erft Health monitoring. Children and young people. By knacks to the barrier?-unlock coiling to lease due. Module 3: Aid for development handicapped children in the District of Rhein-Erft 2006 7 19

[B18] Rhine District of Neuss Children’s health in the Rhine District of Neuss 2005. Current results of new school beginners examination 2005 25 29

[B19] Robert Koch- Institute Contributions to Federal Health Monitoring. Life phase-specific health of children and young people in Germany. Results of the national child and youth health surveys (KiGGS) 2008

[B20] Robert Koch- Institute Contributions to Federal Health Monitoring. Health of children and young people in Schleswig-Holstein 2007 70 71

[B21] Caniato RN Stich HL Alvarenga M Kraemer A Baune BT Changing rates of physical and psychosocial impairments over 9 years in cohorts of school beginners in Germany J Public Health 2008 17 137 144

[B22] Law of Public Health Services (Health Act) from 24 July 2003 Legislative and Law Paper, 452, GNr 2120 1 UG

[B23] Bavarian Law of Education and Instruction (BayEUG) Director ABC 2001

[B24] Stich HL Baune BT Caniato RN AK Associations between preschool attendance and developmental impairments in pre-school children in a six-year retrospective survey BMC Public Health 2006 6 260 10.1186/1471-2458-6-260 17054777 PMC1634854

[B25] Task Force “School and Youth Health in Public Health Services” The school entry examination in 1998

[B26] Tebruegge M Nandini V Ritchie J Does routine child health surveillance contribute to the early detection of children with pervasive developmental disorders? An epidemiological study in Kent, U.K BMC Pediatr 2004 4 4 10.1186/1471-2431-4-4 15053835 PMC375534

[B27] Valtonen R Ahonen T Lyytinen P Lyytinen H Co-occurrence of developmental delays in a screening study of 4-year-old Finnish children Dev Med Child Neurol 2004 46 436 443 15230455 10.1017/s0012162204000726

[B28] Eggers C Psychological irregularities in early school-age Paediatric 1997 3 471 480

[B29] Macha T Petermann F How well form development testing cognitive development?-cognitive dimension of ET 66 German Kinder- und Jugendmedizin 2006 6 381 388

[B30] Kinsbourne M Graf WD Disorders of mental development 2001 6 Philadelphia: Lippincott Williams & Wilkins

[B31] Simeonsson RJ Simeonsson NW Developmental surveillance and intervention 2001 4 St. Louis: Mosby

[B32] Bavarian Health and Food Safety Agency Results of the school receipt investigation to the school year 2004 / 2005. Statistic-epidemiological report 2006

[B33] U.S. Department of Education Early Childhood Longitudinal Study, Birth Cohort (ECLS-B). Preschool- Kindergarten 2007 Psychometric Report 2010

[B34] Ziegert B Neuss A Herpertz-Dahlmann B Kruse W Psychological irregularities of children and young people in general practice. Who needs help? Dt Ärztebl 2002 99 1124 1129

[B35] Stich HL Baune BT Kraemer A Disorders in non-German and German preschool children. Influence of nationality on the individual development 2004 Munich: Juventa

[B36] Esser G Petermann F Developing Diagnostics. Denver Development Scales 2010 Göttinger Bern Wien Paris Oxford Prag Toronto Cambirdge Amsterdam Kopenhagen Stockholm: Hofgrefe

[B37] GA Hv Koletzko B Pediatrics 1997 10 Berlin Heidelberg New York: Springer

[B38] Michaelis R Niemann G Developmental Neurology and Pediatric Neurology 2004 2 Stuttgart: Thieme

[B39] Thompson T Caruso M Ellerbeck K Sex matters in autism and other developmental disabilities J Learn Disabil 2003 36 345 362

[B40] Brody GH Sibling relationship quality: its causes and consequences Annu Rev Psychol 1998 49 1 24 10.1146/annurev.psych.49.1.1 9496619

[B41] Azmitia M Hesser J Why siblings are important agents of cognitive development: A comparison of siblings and peers Child Development 1993 64 430 444 10.2307/1131260

[B42] Minnett AM Vandell DL Santrock JW The effects of sibling status on sibling interaction: influence of birth order, age spacing, sex of child, and sex of sibling Child Development 1983 54 1064 1072 10.2307/1129910

